# Can ChatGPT-5 educate the public about vasectomy?: a Google Trends–based expert panel assessment

**DOI:** 10.3389/fdgth.2026.1726517

**Published:** 2026-03-18

**Authors:** Ali C. Albaz, Oğuzcan Erbatu, Okan Yiğit, Oktay Üçer, Gökhan Temeltaş, Talha Müezzinoğlu

**Affiliations:** Department of Urology, Faculty of Medicine, Manisa Celal Bayar Universitesi, Manisa, Türkiye

**Keywords:** artificial intelligence, ChatGPT-5, health communication, patient education, public health, vasectomy

## Abstract

**Background:**

ChatGPT-5, the latest multimodal large language model (LLM), has gained remarkable public attention for its ability to provide real-time and context-aware health information. However, its effectiveness in addressing sensitive urological topics such as vasectomy has not been systematically evaluated.

**Objective:**

This study aimed to evaluate the accuracy, completeness and public suitability of ChatGPT-5's responses to frequently asked questions about vasectomy, derived from Google Trends data reflecting real-world public interest.

**Methods:**

A total of eight experts—four urologists, two public health specialists, one obstetrician-gynecologist and one fertility nurse—independently assessed ChatGPT-5's responses to ten high-frequency vasectomy-related questions. Each response was rated using six 5-point Likert-scale criteria: medical accuracy, completeness, clarity, tone, public usefulness and recommendability. Descriptive statistics, Kruskal–Wallis tests and two-way random-effects intraclass correlation coefficients (ICC, 95% CI) were applied for statistical analysis.

**Results:**

The mean ratings across evaluation domains ranged from 3.75 to 4.04. Clarity of language and tone appropriateness received the highest scores, whereas medical accuracy and comprehensiveness demonstrated greater dispersion. No statistically significant differences were observed among expert subgroups (*p* > 0.05). Inter-rater reliability was very low (ICC = −0.01), indicating substantial variability across expert evaluations.

**Conclusions:**

In this exploratory assessment, ChatGPT-5 responses to vasectomy-related public questions were frequently perceived as clear and appropriately framed for informational use. However, variability across expert ratings and the absence of layperson validation underscore the need for cautious interpretation. Large language model outputs may serve as supportive educational resources when accompanied by expert oversight and audience-specific adaptation.

## Introduction

The rapid evolution of artificial intelligence (AI) has reshaped how the public encounters medical knowledge, accelerating a shift from static texts to interactive, conversation-based explanations. Since the release of ChatGPT, large language models (LLMs) have demonstrated the capacity to parse complex clinical information and articulate it in accessible prose; performance on examination-style assessments such as the USMLE and advanced national licensing tests has further fueled interest in educational and patient-facing applications (1, 2). Beyond scorecards, early feasibility work and broader syntheses suggest growing opportunities for LLMs across clinical and research scenarios, including medical pedagogy and communication (3, 4). Parallel commentary has highlighted pragmatic gains in scientific writing workflows, while also warning that editorial shortcuts can mask conceptual gaps if expert checks are omitted (5). As the technology and its prompting practices mature exemplified by structured frameworks for safe, purpose-fit prompting in clinical contexts the question is no longer whether LLMs can speak medicine, but whether they can do so reliably for the public (6).

That reliability is the crux. Even when language is polished, outputs may omit key qualifiers, overgeneralize, or present confident errors. Systematic and narrative accounts in health education repeatedly flag this risk and call for transparent oversight (3, 4). The men's health domain illustrates the stakes: misinformation circulating through social media and AI-mediated channels can nudge decisions in ways that diverge from evidence, underscoring the need for professional guardrails and verifiable sourcing (7). Clinicians’ own views are mixed across specialties enthusiasm for efficiency tempered by concerns about accuracy, scope creep and medico-legal responsibility (8). Small applied studies likewise report uneven performance when LLMs draft clinical documents, with readability often high but factual precision variable, reinforcing the need for domain-expert review before dissemination (9).

Vasectomy provides a particularly instructive use-case. It is a safe, effective and permanent method of male contraception, yet remains underutilized relative to its benefits (13–15). Misconceptions persist fear of sexual dysfunction, masculinity concerns, and perceived irreversibility amplified by stigma and patchy counseling access (13, 14). Obstetrician-gynecologists are well-positioned to normalize counseling and address reproductive decision-making within couples, but opportunities are frequently missed (14). Population-level evidence suggests disparities in utilization by demographic group, highlighting informational inequities that digital tools—including generative AI—might either mitigate or entrench (15). In this environment, an LLM that is clear, calm in tone and accurate could plausibly improve public understanding; the same tool, if imprecise, could magnify confusion.

Early evaluations specific to vasectomy reflect this tension. Public-facing question-and-answer (Q&A) studies report that LLM responses often read well and are framed appropriately, yet show variable completeness and medical accuracy across items, prompting cautions about unsupervised use (10, 11). A contemporaneous commentary reiterated this need for expert oversight when deploying chatbots for sensitive urological topics, emphasizing transparent sourcing and error correction pathways (12). Outside urology, cross-disciplinary appraisals in otolaryngology and dermatology show similar patterns strong readability and efficiency with inconsistent factual depth again pointing to the centrality of clinician review for patient-facing content (8, 9). Meanwhile, broader clinical narratives (e.g., emergency-department sepsis) catalog expanding roles for AI while advocating rigorous validation and context-specific safeguards (16). Importantly, evidence from pediatric surgical education suggests that LLMs can facilitate patient education when embedded within supervised workflows that prioritize empathy, clarity and alignment with guidelines (17).

Against this backdrop, the present study examines ChatGPT-5 a multimodal successor with real-time, context-aware capabilities in the specific, socially sensitive domain of vasectomy. Using Google Trends to surface the ten most frequently searched public questions, we subjected model outputs to structured appraisal by a multidisciplinary expert panel, rating medical accuracy, completeness, clarity, tone, usefulness and recommendability. Our goal is not to adjudicate a binary “safe or unsafe” verdict, but to characterize performance where it matters most: the intersection of correctness and comprehensibility for lay readers. In doing so, we build on prior licensing-exam performance (1, 2), feasibility analyses (3) and health-education syntheses (4), while addressing documented risks of misinformation in men's health (7) and known counseling gaps around vasectomy (13, 14). The resulting profile of strengths and limitations aims to inform practical deployment: where ChatGPT-5 can responsibly augment public education under expert supervision and where additional guardrails, prompt design, or human curation remain essential.

## Materials and methods

This descriptive, cross-sectional study evaluated the quality of ChatGPT-5 outputs for public education on vasectomy. The primary objective was to quantify model performance across six domains medical accuracy, completeness, clarity, tone appropriateness, usefulness for general audiences and recommendability as general health information. Secondary objectives were to (i) characterize readability of the same outputs, (ii) explore between-question and between-specialty variation in ratings and (iii) estimate inter-rater reliability.

### Question identification

To reflect real-world information-seeking behavior, we used Google Trends with the query term “vasectomy” set to Worldwide, Past 12 months and accessed in August 2025. “Top” and “Related queries” lists (i.e., the most frequently searched queries and algorithmically associated search terms provided by Google Trends) were exported as CSV files and time-stamped screenshots were archived. From the top 20 items, two investigators independently removed duplicates, semantic near-duplicates, navigational or commercial queries, and items with low informational value; discrepancies were resolved by a third reviewer. The final dataset comprised 10 unique, high-volume questions. Items were lightly edited to preserve natural public phrasing without altering meaning. All questions were expressed in plain natural language and formatted as direct layperson-style inquiries. Original and edited versions were archived side-by-side.

### Model and interaction protocol

All answers were generated with ChatGPT-5 (OpenAI; multimodal LLM, paid access) via the ChatGPT web interface in August 2025. Each question was submitted in a fresh session as a single-turn user prompt; no follow-up prompts were used and no chain-of-thought content was requested. Vendor default decoding and safety settings were maintained (temperature/top-p/max_tokens left at defaults) and no external tools or web browsing were enabled. A single candidate (*n* = 1) was requested per prompt; no random seed was set. Prompts were frozen *a priori* and are provided verbatim in Supplementary Table 1 (system prompt and user-prompt template). Outputs were time-stamped (UTC+3) and archived without manual editing before rating.

### Expert panel, eligibility and blinding

Eight independent evaluators rated the answers under blinded conditions (no model/version metadata visible): Urology (two professors, two assistant professors), Public Health (one professor, one assistant professor), Obstetrics and Gynecology (one professor) and Reproductive Nursing (one fertility nurse). Eligibility criteria included ≥5 years post-training, a current academic appointment and absence of financial or institutional ties to the model vendor. Before scoring, all raters reviewed a one-page rubric with domain definitions and anchor examples. The order of the 10 questions was randomized for each rater to minimize potential order effects. Ratings were entered individually into a secure form; no missing data occurred and no imputation was required.

### Scoring domains

Raters used a 5-point Likert scale (1 = completely inadequate; 5 = completely adequate) for the following domains: (1) medical accuracy (evidence-concordant, clinically sound); (2) completeness (presence of key elements without critical omissions); (3) clarity/structure (plain language, logical organization); (4) tone/language appropriateness (neutral, respectful, non-alarmist); (5) usefulness for general audiences (understandable and actionable for lay readers); and (6) recommendability (safely shareable as general health information). Domain definitions and anchors are provided in Supplementary Table 2.

### Readability assessment

Readability of each archived answer was quantified on the English-language outputs using the Flesch Reading Ease Score (FRES; 0–100, higher scores indicating easier readability) and the Flesch–Kincaid Grade Level (FKGL; U.S. grade level). These metrics are widely used and validated tools in health communication research to estimate textual accessibility for lay audiences. Metrics were computed on the exact evaluated texts via readabilityformulas.com on the same day as expert scoring; calculation logs and outputs were archived. Expert ratings were collected using a 5-point Likert scale, a standard and validated approach for structured expert-based assessments of qualitative attributes such as accuracy, clarity, completeness, and usefulness.

### Statistical analysis

For each domain, scores were summarized as means (±SD) overall, by question and by specialty. Between-question differences were tested using Kruskal–Wallis as the primary non-parametric approach; when assumptions were tenable (normality by Shapiro–Wilk; homogeneity by Levene), one-way ANOVA was reported as a sensitivity analysis. Between-specialty differences were assessed using one-way ANOVA (Shapiro–Wilk for normality; Levene for homogeneity). When assumptions were not met, we used the Kruskal–Wallis test. For multiple comparisons we applied Tukey HSD (ANOVA) or Dunn tests with Holm correction (Kruskal–Wallis). Inter-rater reliability was estimated with ICC(2,k) (two-way random-effects, absolute agreement) with 95% confidence intervals. Readability–rating associations (e.g., FRES with clarity/usefulness) were examined exploratorily using Spearman's *ρ*. All tests were two-sided with *α* = 0.05. Analyses were performed in GraphPad Prism v10.1.0 (San Diego, CA, USA). All analysis decisions were finalized *a priori* and time-stamped in an internal log.

### Ethics and AI-use disclosure

This study was approved by the Ethics Committee of the Faculty of Medicine, Manisa Celal Bayar University (approval date: 16 July 2025; approval no.: 20.478.476/3299). No patient-level data were collected. All expert participants provided written informed consent prior to participation.

ChatGPT-5 was used only to generate the answers under evaluation. Study design, statistical analyses and all manuscript text including verification of the literature, were authored and manually validated by the investigators using PubMed-indexed sources. No patient information was provided to the model and no generative system assisted with data analysis or reference validation.

## Results

**Figure 1:**
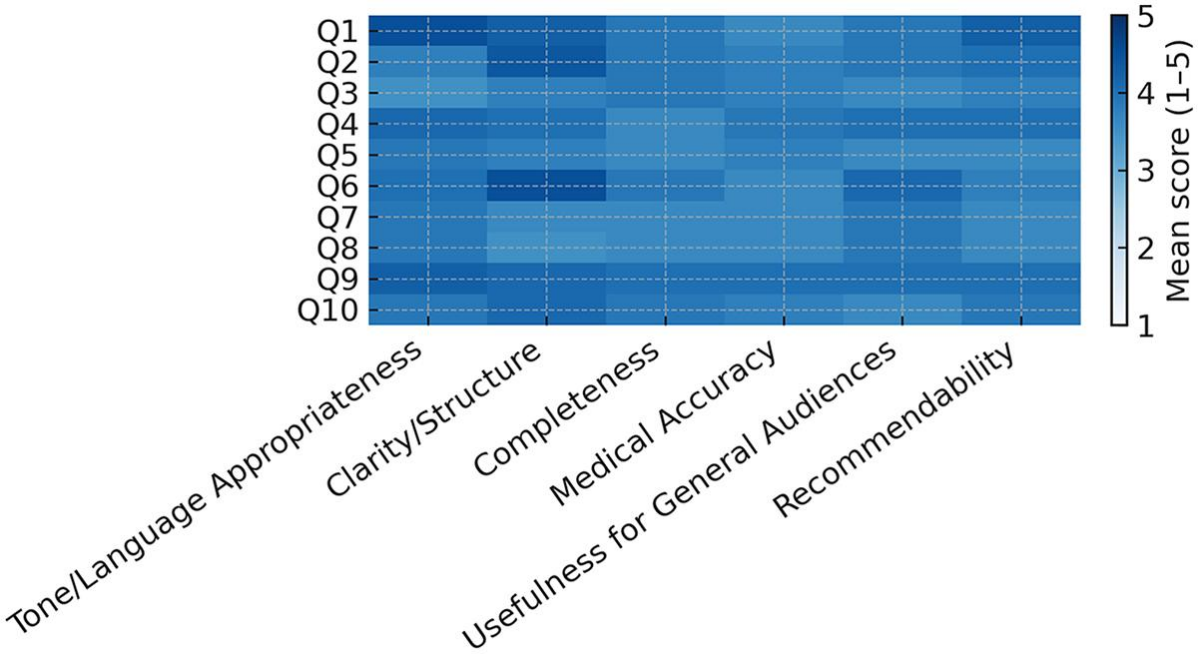
Heatmap of mean Likert scores (1–5) for each of the 10 public questions (rows) across six domains (columns; fixed order: *tone/language appropriateness, clarity/structure, completeness, medical accuracy, usefulness for general audiences, recommendability*). A single sequential color scale (legend 1–5; lighter = higher) highlights higher central tendencies in Clarity/Structure and Tone/Language Appropriateness, with comparatively lower means more frequent in Medical Accuracy and Completeness. Numeric values are intentionally omitted; corresponding statistics are reported in Table 1 and Supplementary Table 2.

**Figure 2:**
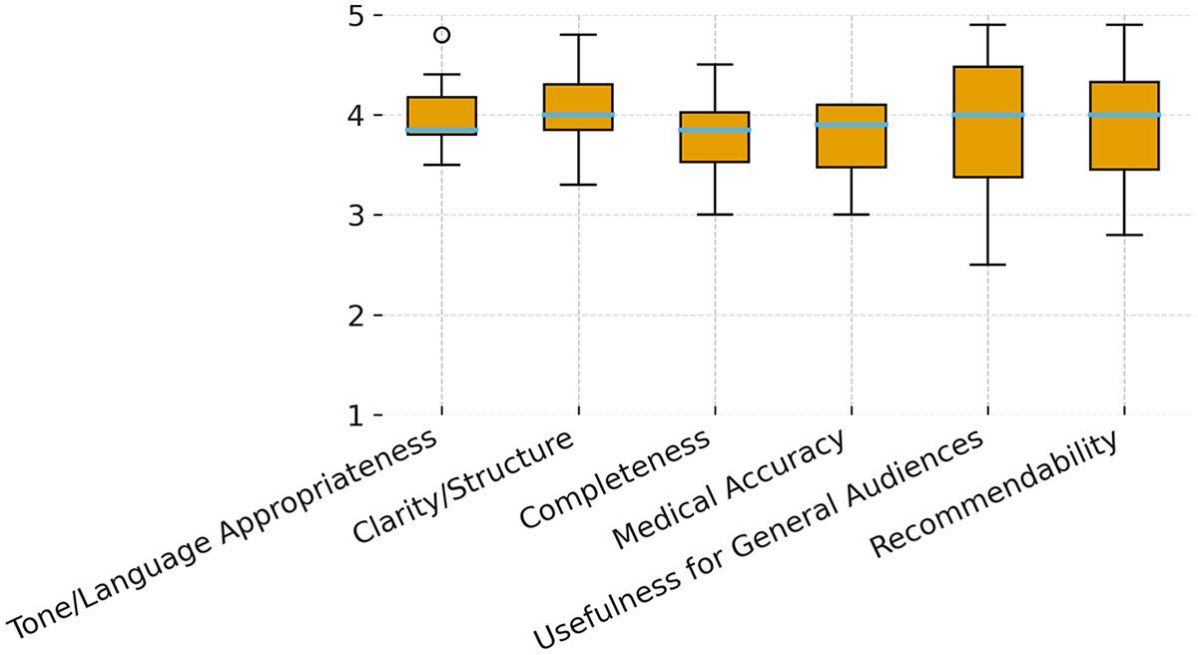
Box-and-whisker plots of expert ratings by domain (Likert 1–5; *n* = 8 experts). Center lines indicate medians; boxes show the interquartile range (IQR); whiskers extend to 1.5 × IQR; outliers are plotted as points. The *y*-axis spans 1–5. Distributions are wider in Recommendability and Usefulness for General Audiences, indicating greater between-expert variability and narrower for tone/language appropriateness and clarity/structure.

We analyzed 10 public questions derived from Google Trends. For each question, ChatGPT-5 generated a single answer that was independently rated by eight experts across six domains on a 5-point Likert scale (1 = completely inadequate; 5 = completely adequate). The rating matrix was complete with no missing observations. Expert-level and pooled summary statistics are presented in Table 1; the question-by-domain pattern appears in Figure 1 (heatmap) and domain-wise rating distributions in Figure 2 (boxplots).

**Table 1:** Mean (±SD) scores by domain and expert (Likert 1–5), plus an overall mean (±SD) row.

Expert	Tone/language appropriateness	Clarity/structure	Completeness	Medical accuracy	Usefulness for general audiences	Recommendability
Prof Uro 1	3.50	3.30	3.30	3.50	3.00	3.30
Prof Uro 2	3.80	4.30	3.00	3.00	4.00	4.20
Prof ObGyn	4.40	3.90	4.10	4.10	2.50	2.80
Prof PublicHealth	3.80	4.00	3.80	4.10	4.70	4.90
AsstProf Uro1	3.80	3.70	3.60	3.40	3.50	3.50
AsstProf Uro2	4.10	4.00	4.00	4.10	4.40	4.10
AsstProf PublicHealth	4.80	4.80	4.50	4.00	4.90	4.70
Fertility nurse	3.90	4.30	3.90	3.80	4.00	3.90
Overall mean (±SD)	4.01 ± 0.41	4.04 ± 0.45	3.77 ± 0.47	3.75 ± 0.41	3.88 ± 0.83	3.92 ± 0.71

Likert scale: 1 = completely inadequate; 5 = completely adequate. *n* = 10 questions × 8 raters; no missing data. Fixed domain order: tone/language appropriateness, clarity/structure, completeness, medical accuracy, usefulness for general audiences, recommendability.

Across all raters and questions, mean scores ranged from 3.75 to 4.04. Central tendencies were highest for Clarity/Structure (overall 4.04 ± 0.45) and Tone/Language Appropriateness (4.01 ± 0.41). Although still above the scale midpoint, Medical Accuracy and Completeness showed comparatively broader dispersion 3.75 ± 0.41 and 3.77 ± 0.47, respectively while Usefulness for General Audiences and Recommendability averaged 3.88 ± 0.83 and 3.92 ± 0.71. Consistent with these aggregates, Figure 1 shows lighter cells (higher means) clustering in the Clarity and Tone columns, whereas darker cells (lower means) are more frequent in the Accuracy and Completeness columns, indicating variability in inclusion of procedural nuance and content depth across questions.

Between-specialty comparisons using Kruskal–Wallis revealed no statistically significant differences for any domain (*p* > 0.05). The corresponding H statistics and exact two-sided *p* values for each domain are reported in Supplementary Table 2. Overall inter-rater agreement was poor, with ICC(2,k) = –0.01; the 95% confidence interval provided in Supplementary Table 3 is consistent with poor agreement. Distributional features in Figure 2 (wider IQRs and occasional outliers, especially for Medical Accuracy and Completeness) visually reflect heterogeneous rating patterns despite favorable central tendencies. Readability analyses (Flesch Reading Ease and Flesch–Kincaid Grade Level) indicated lay-readable prose overall; readability trends qualitatively paralleled higher Clarity/Structure ratings but did not imply sufficiency in Medical Accuracy or Completeness. Per-question readability summaries and calculation logs appear in Supplementary Table 3. Where distributional assumptions were tenable, one-way ANOVA summaries did not alter conclusions drawn from the primary non-parametric analyses; leave-one-group-out checks (excluding each specialty in turn) did not materially change pooled means or the ICC estimate. No imputation was required and no multiplicity adjustment was applied given the exploratory domain-wise framework.

## Discussion

Public-facing use of LLMs is accelerating across health communication. However, determining how model-generated answers should be positioned as reliable and patient-safe educational resources — particularly for stigmatized topics such as vasectomy — remains an open question. Vasectomy is a safe yet underused method and the online information environment around men's health is vulnerable to misinformation; together these factors make scalable, high-quality education both necessary and difficult (18, 19). In this context, we evaluated ChatGPT-5 responses to frequently asked public questions about vasectomy using a multidisciplinary panel and a six-domain rubric.

Overall, ratings favored clarity of language, tone appropriateness and perceived usefulness for lay audiences, while medical accuracy and comprehensiveness showed broader dispersion. This pattern is consistent with prior observations that LLM outputs often strike a readability–granularity trade-off: linguistically polished, empathetic text may under-specify procedural details that clinicians consider “minimum safe content” for risk communication [e.g., failure/recanalization framing, post-vasectomy semen analysis (PVSA) requirements and timing] (20, 21).

Although mean scores did not differ significantly between specialties, inter-rater reliability was very low (ICC = −0.01), indicating substantial variability across expert evaluations and limiting the interpretability of aggregated ratings. Between-specialty comparisons were not significant on ANOVA and Kruskal–Wallis analyses, and adjusted *post-hoc* contrasts (Tukey HSD/Dunn–Holm) revealed no significant pairwise differences. In practice, specialties appeared to weight different evaluative dimensions: urologists emphasized procedural specificity and PVSA logistics, whereas public-health and obstetrics-gynecology experts prioritized framing, actionability, and tone. The widened interquartile ranges observed for medical accuracy and comprehensiveness (Figure 2) illustrate this heterogeneity. Methodologically, negative or near-zero ICC values indicate substantial rater-by-criterion variability and warrant caution when interpreting central tendencies without explicit dispersion metrics and structured rater guidance (22). Two system-level considerations contextualize these findings. First, hallucination — the generation of fluent but incorrect statements — remains a recognized failure mode that may subtly compromise health-related information. Human verification is therefore essential, particularly for behavior-influencing claims (23). Second, prompt sensitivity and framing effects can shift emphasis (e.g., complications vs. sexual function) or introduce commercial/ideological cues, underscoring the need for transparent provenance and content accountability (24). In public-facing deployments, we therefore recommend: version and date stamps for all outputs; retrieval-anchored on-page citations where feasible; explicit “not medical advice” and escalation cues (“seek care if…”); and mandatory clinician review for high-impact statements.

Vasectomy-specific considerations are threefold. First, myth correction must balance reassurance and uncertainty particularly around sexual function, long-term risks and expectations about reversibility (17). Second, operational details should be conspicuous and standardized (PVSA necessity and timing windows, typical convalescence and restrictions), as burying these items invites over-trust in simplified narratives (25). Third, equity matters: utilization disparities by sociodemographic strata persist, so public materials should be culturally adapted, cost-aware and clearly signpost to local services (9).

Our findings align with prior evaluations of ChatGPT-based systems in the field of andrology. In a recent study assessing ChatGPT's performance in explaining andrological diseases to patients, Ergin and Sancı reported that AI-generated responses were generally readable and accessible, but that completeness and clinically relevant detail varied across topics (26). Similarly, we observed strong performance in clarity and tone for vasectomy-related questions, accompanied by greater dispersion in medical accuracy and comprehensiveness. Together, these findings suggest that while large language models can effectively lower barriers to initial patient understanding in men's reproductive health, expert oversight remains essential to ensure content sufficiency and patient safety in public-facing educational use.

Strengths of this study include a multidisciplinary rater panel, a six-domain structured rubric tailored to public-health communication needs, real-world question selection driven by search interest and complementary parametric/non-parametric analyses with visualization of dispersion. Limitations include a modest expert sample; exclusion of lay participants (limiting external validity for comprehension and trust outcomes); English-only evaluation; a single-model, cross-sectional design that precludes head-to-head comparisons and model-drift tracking; and residual prompt-variation sensitivity. In addition, the evaluation was limited to the paid version of ChatGPT-5, and response characteristics may differ in free-access versions available to the general public. The very low inter-rater reliability indicates substantial variability in expert evaluations and constrains the interpretability of aggregated scores. This variability may partly reflect differences in disciplinary perspectives and evaluative thresholds, although its precise sources cannot be determined within the present design. These limitations may be addressed in future studies through larger cross-cultural samples, inclusion of lay panels with literacy stratification, pre-registered multi-model protocols, comparative model designs, and scheduled reassessments following model updates.

### Implications

Within a clear governance framework, ChatGPT-5 may have a potential role as a top-of-funnel educational adjunct for vasectomy lowering the threshold for first-contact information seeking provided outputs are retrieval-grounded, date-stamped, clinically audited and embedded in explicit escalation pathways. A Delphi-style, cross-disciplinary minimum safe content checklist [e.g., numerical ranges with uncertainty for failure risk; explicit post-vasectomy semen analysis (PVSA) requirements and timing; standard “when to seek care” cues] could improve rater alignment and enhance inter-rater reliability. Finally, although benchmark results indicate improving general competence in recent models, exam-style performance does not equal public-education sufficiency; dispersion patterns in our data show that mean adequacy does not imply consensus and oversight remains essential (1, 7, 27).

## Conclusion

In this exploratory multidisciplinary assessment, ChatGPT-5 generated vasectomy-related responses that were often judged to be clear and appropriately framed for public-facing informational use. Nevertheless, the marked variability across expert ratings, together with the very low inter-rater agreement, indicates that evaluations of AI-generated medical content remain highly dependent on individual interpretation.

These observations reinforce that large language model outputs should not be viewed as independent clinical guidance, particularly in sensitive areas of men's reproductive health. Rather, such systems may serve as supportive educational resources when accompanied by appropriate expert review and contextualization.

Considering the exploratory design and the limited number of evaluated questions, the findings warrant cautious interpretation. Further studies using expanded datasets, refined evaluation frameworks, and layperson-centered perspectives are needed to clarify the consistency, reliability, and educational relevance of AI-generated health information.

## Data Availability

The original contributions presented in the study are included in the article/Supplementary Material, further inquiries can be directed to the corresponding author.

## References

[1] Kung TH Cheatham M Medenilla A Sillos C De Leon L Elepaño C Performance of ChatGPT on USMLE: potential for AI-assisted medical education using large language models. PLOS Digit Health. (2023) 2(2):e0000198. 10.1371/journal.pdig.0000198 36812645 PMC9931230

[2] Cascella M Montomoli J Bellini V Bignami E. Evaluating the feasibility of ChatGPT in healthcare: an analysis of multiple clinical and research scenarios. J Med Syst. (2023) 47(1):33. 10.1007/s10916-023-01925-4 36869927 PMC9985086

[3] Sallam M. ChatGPT utility in healthcare education, research and practice: systematic review on the promising perspectives and valid concerns. Healthcare (Basel). (2023) 11(6):887. 10.3390/healthcare11060887 36981544 PMC10048148

[4] Salvagno M Taccone FS Gerli AG. Can artificial intelligence help for scientific writing? Crit Care. (2023) 27(1):75. 10.1186/s13054-023-04380-2 36841840 PMC9960412

[5] Lin SY Chan PK Hsu WH Kao CH. Exploring the proficiency of ChatGPT-4: an evaluation of its performance in the Taiwan advanced medical licensing examination. Digit Health. (2024) 10:20552076241237678. 10.1177/20552076241237678 38449683 PMC10916498

[6] Boit S Patil R. A prompt engineering framework for large language model-based mental health chatbots: design principles and insights for AI-supported care. JMIR Ment Health. (2025). 10.2196/75078 41202205 PMC12594504

[7] Loeb S Sengupta S Butaney M Macaluso JN. The rise of misinformation in men’s health: social media, AI, and the need for professional oversight. Eur Urol Focus. (2023) 9(2):435–9. 10.1016/j.euf.2022.12.00636577611

[8] Patel J Nguyen BT. Vasectomy: an opportunity for obstetricians and gynecologists. Clin Obstet Gynecol. (2020) 63(2):289–94. 10.1097/GRF.0000000000000520 31876637

[9] Eisenberg ML Henderson JT Amory JK Smith JF Walsh TJ. Racial differences in vasectomy utilization in the United States: data from the national survey of family growth. Urology. (2009) 74(5):1020–4. 10.1016/j.urology.2009.06.042 19773036 PMC2784091

[10] Carlson JA Cheng RZ Lange A Nagalakshmi N Rabets J Shah T Accuracy and readability of artificial intelligence chatbot responses to vasectomy-related questions: public beware. Cureus. (2024) 16(8):e67996. 10.7759/cureus.67996 39347335 PMC11427961

[11] Asokan A Massey CJ Tietbohl C Kroenke K Morris M Ramakrishnan VR. Physician views of artificial intelligence in otolaryngology and rhinology: a mixed methods study. Laryngoscope Investig Otolaryngol. (2023) 8(6):1468–75. 10.1002/lio2.1177 38130265 PMC10731489

[12] Farooq F Cooper H Shipman A Michell CD. Artificial intelligence versus traditional method in generating dermatology consultation letters: a pilot study comparing accuracy, readability and efficiency. Clin Exp Dermatol. (2025):llaf323. 10.1093/ced/llaf323 40674470

[13] Almidani O Alhosani H Raheem OA. Commentary on: can AI chatbots accurately answer patient questions regarding vasectomies? Int J Impot Res. (2024). 10.1038/s41443-024-00982-8 39313568

[14] Mouhawasse E Haff CW Kumar P Lack B Chu K Bansal U Can AI chatbots accurately answer patient questions regarding vasectomies? Int J Impot Res. (2024). 10.1038/s41443-024-00970-y 39179908

[15] Uddin J Feng C Xu J. Health communication on the internet: promoting public health and exploring disparities in the generative AI era. J Med Internet Res. (2025) 27:e66032. 10.2196/66032 40053755 PMC11926442

[16] Bryson X Albarran M Pham N Salunga A Johnson T Hogue GD Artificial intelligence-based large language models can facilitate patient education. J Pediatr Soc North Am. (2025) 12:100196. 10.1016/j.jposna.2025.100196 40791971 PMC12337203

[17] Shih G Turok DK Parker WJ. Vasectomy: a safe and underutilized contraceptive method. Am J Obstet Gynecol. (2021) 224(4):352–7. 10.1016/j.ajog.2020.08.049

[18] Black A Guilbert E Costescu D Dunn S Fisher W Kives S Canadian contraception consensus (part 1 of 4). J Obstet Gynaecol Can. (2015) 37(10):936–42. 10.1016/s1701-2163(16)30033-0 26606712

[19] Black A Guilbert E Costescu D Dunn S Fisher W Kives S Canadian contraception consensus (part 2 of 4). J Obstet Gynaecol Can. (2015) 37(11):1033–9. 10.1016/s1701-2163(16)30054-8 26629725

[20] Ferrari-Light D Merritt RE Souza D Ferguson D Harrison MK Madariaga S Evaluating ChatGPT as a patient resource for frequently asked questions about lung cancer surgery – a pilot study. J Thorac Cardiovasc Surg. (2025) 169(4):1174–84.e18. 10.1016/j.jtcvs.2024.09.030 39326732

[21] Whiles BB Bird VG Canales BK DiBianco JM Terry RS. Caution! AI bot has entered the patient chat: chatGPT has limitations in providing accurate urologic healthcare advice. Urology. (2023) 180:278–84. 10.1016/j.urology.2023.07.010 37467806

[22] Koo TK Li MY. A guideline of selecting and reporting intraclass correlation coefficients for reliability research. J Chiropr Med. (2016) 15(2):155–63. 10.1016/j.jcm.2016.02.012 27330520 PMC4913118

[23] Yi Y Kim KJ. The feasibility of using generative artificial intelligence for history taking in virtual patients. BMC Res Notes. (2025) 18(1):80. 10.1186/s13104-025-07157-8 39994780 PMC11849343

[24] Rao VS Kumar A Lakkaraju H Shah NB. Detecting LLM-generated peer reviews. PLoS One. (2025) 20(9):e0331871. 10.1371/journal.pone.0331871 40982562 PMC12453209

[25] Agarwal A Gupta S Sharma RK Finelli R Kuroda S Vij SC Post-vasectomy semen analysis: optimizing laboratory procedures and test interpretation through a clinical audit and global survey of practices. World J Mens Health. (2022) 40(3):425–41. 10.5534/wjmh.210191 35021311 PMC9253792

[26] Ergin IE Sancı A. Can ChatGPT help patients understand their andrological diseases? Rev Int Androl. (2024) 22(2):14–20. 10.22514/j.androl.2024.010 39135370

[27] Paluszek O Loeb S. Artificial intelligence and patient education. Curr Opin Urol. (2025) 35(3):219–23. 10.1097/MOU.0000000000001267 39945126 PMC11964839

