# Supplementary material for: Can ChatGPT-5 educate the public about vasectomy?: a Google Trends–based expert panel assessment
Source: Front Digit Health. 2026 Mar 18;8:1726517. doi: 10.3389/fdgth.2026.1726517 (PMC13040449; doi:10.3389/fdgth.2026.1726517)
Supplement: Supplementary file 3 [file Table3.docx]

# Supplementary Table A3. Readability metrics of ChatGPT‑5 responses to vasectomy‑related public questions

| Question | Flesch Reading Ease | Flesch–Kincaid Grade Level |
| --- | --- | --- |
| Q1 | 43.9 | 11.0 |
| Q2 | 45.2 | 11.0 |
| Q3 | 23.5 | 14.0 |
| Q4 | 53.2 | 9.0 |
| Q5 | 46.6 | 9.7 |
| Q6 | 28.7 | 13.0 |
| Q7 | 50.1 | 10.2 |
| Q8 | 48.4 | 9.3 |
| Q9 | 57.8 | 7.5 |
| Q10 | 44.0 | 11.3 |

Flesch Reading Ease ranges from 0–100 (higher scores indicate easier readability). Flesch–Kincaid Grade Level represents the U.S. school grade required to understand the text.
